# Effects of cochlear implantation on quality of life in patients with age-related hearing loss: a systematic review

**DOI:** 10.3389/fnins.2026.1778985

**Published:** 2026-02-25

**Authors:** Xiangke Wang, Hongxia Lan, Ruilin Li, Zhanhang Zheng, Chenxingzi Wu, Shuhong Qin, Wenjuan Wang, Ting Chen

**Affiliations:** 1 School of Nursing, Guangxi University of Chinese Medicine, Nanning, Guangxi, China; 2 The Second People's Hospital of Foshan, Foshan, Guangdong, China; 3 Jiangbin Hospital of Guangxi Zhuang Autonomous Region, Nanning, Guangxi, China

**Keywords:** age-related hearing loss, cochlear implantation, health-related quality of life, quality of life, systematic review

## Abstract

**Background:**

Age-related hearing loss (ARHL) is among the most prevalent sensory impairments in older adults. However, the magnitude and time course of quality-of-life (QoL) gains associated with cochlear implantation (CI) in ARHL, as well as potential differences across older age strata, have not been synthesized systematically. We conducted a systematic review to characterize QoL changes after CI in ARHL and to contrast the responsiveness of hearing/CI-specific instruments with that of generic QoL measures.

**Methods:**

Following PRISMA, we searched PubMed, Web of Science, Embase, the Cochrane Library, and CNKI from inception to 7 December 2025. Eligible studies enrolled adults aged ≥60 years meeting an ARHL definition, receiving CI, and reporting outcomes from validated QoL instruments. Two reviewers independently performed study selection and data extraction. Risk of bias in non-randomized studies was assessed using the ROBINS-I tool. Outcomes measured by different types of QoL instruments at each follow-up time point were synthesized narratively.

**Results:**

We included 16 studies (842 participants). According to ROBINS-I, most studies had at least moderate risk of bias, and approximately one third were judged to have a serious risk of bias. Across studies, hearing/CI-specific measures (e.g., Nijmegen Cochlear Implant Questionnaire, NCIQ and Glasgow Benefit Inventory, GBI) more consistently detected post-CI gains, particularly in domains related to sound perception and social support. In contrast, generic QoL instruments (e.g., WHOQOL-OLD) more often suggested a delayed benefit signal, typically becoming statistically apparent around 6 months after surgery and mainly involving sensory abilities, social participation, and mental health, while changes in physical-functioning domains were limited.

**Conclusion:**

On the basis of observational studies with predominantly moderate to serious risk of bias, current evidence suggests that QoL tends to improve after CI in adults with ARHL and that chronological age itself does not appear to be a major constraint on CI-related benefit. Future studies should prioritize CI-specific instruments (e.g., Cochlear Implant Quality of Life, CIQOL), harmonize follow-up schedules, and explicitly examine longer-term benefit trajectories in the oldest-old to strengthen evidence for expectation management and rehabilitation planning.

## 1 Introduction

With accelerating global population aging, age-related hearing loss (ARHL) has become one of the most prevalent sensory impairments in older adults (Tsai Do et al., 2024). According to the World Health Organization (WHO), the prevalence of ARHL increases steeply with age, from 10.9–17.6% at 60–69 years to 23.4–31.3% at 70–79 years, 41.9–51.2% at 80–89 years, and 52.9–64.9% among adults aged ≥90 years (World report on hearing, 2021). ARHL is associated not only with difficulties in speech understanding and listening fatigue but also with restricted social participation, loneliness, social isolation, and depressive symptoms. Loneliness and social isolation themselves are recognized by the WHO as major risk factors for mental health problems in later life (Ying et al., 2023; Bai et al., 2025; Li et al., 2025). Thus, interventions for ARHL have value beyond restoring auditory function, with the potential to enhance overall quality of life (QoL) by improving communication and social participation.

For adults with moderate-to-profound ARHL, cochlear implantation (CI) is widely regarded as one of the most effective options for auditory rehabilitation. Accumulating evidence indicates that advanced age per se is not an absolute contraindication to CI, and that older adults with multiple chronic conditions can still achieve meaningful gains in speech recognition and self-reported benefit (Bocquillon et al., 2025; Zhan et al., 2025). Nevertheless, concerns about CI outcomes in older adults persist in clinical practice. Conventional audiologic metrics capture only part of the changes that matter to patients, including communication, social participation, and emotional well-being (Brumer et al., 2022; Rasmussen et al., 2022; Philpott et al., 2025). In addition, expectations about postoperative benefit, the process of adaptation, and the availability of rehabilitation support are likely to shape subjective QoL outcomes (McRackan et al., 2021b).

QoL is a multidimensional construct centered on individuals’ subjective experience, encompassing auditory experiences, social interactions, mental health, and overall perceived health (Skevington et al., 2004). In CI research, hearing/CI-focused condition- or intervention-specific questionnaires (hereafter “hearing/CI-specific instruments”), such as the Nijmegen Cochlear Implant Questionnaire (NCIQ) and the Glasgow Benefit Inventory (GBI), primarily target auditory and social functioning and are considered more sensitive to CI-related benefit (Robinson et al., 1996; Hinderink et al., 2000). By contrast, generic or health-related QoL questionnaires (hereafter “generic instruments”), such as the World Health Organization Quality of Life–OLD module (WHOQOL-OLD), the Health Utilities Index (HUI2/HUI3), and the Short Form Health Survey (SF-36), capture broader health status and functional independence, and their patterns and sensitivity of change do not fully align with hearing-specific measures (McHorney et al., 1994; Feeny et al., 2002; Power et al., 2005). Existing studies also vary widely in follow-up duration, ranging from several months to many years after surgery, and QoL improvements appear to be time dependent, complicating direct comparisons across studies.

Although prior reviews have examined overall outcomes of CI in older adults (Andries et al., 2021), they often pool heterogeneous etiologies of age-related hearing loss and devote limited attention specifically to ARHL. They also have not systematically contrasted the benefit patterns captured by hearing-specific versus generic QoL instruments across different follow-up time points. Against this background, we conducted a systematic review to synthesize evidence on QoL changes after CI in adults with ARHL, with three objectives: (1) to characterize overall QoL changes after CI in ARHL; (2) to describe the benefit profiles detected by hearing/CI-specific versus generic instruments at different follow-up time points; and (3) to explore potential differences in the magnitude of QoL improvement across older age strata. Our aim is to inform expectation management, the timing of follow-up assessments, and the selection of outcome measures for CI in ARHL.

## 2 Methods

### 2.1 Data sources and search strategy

This systematic review was conducted in accordance with the Preferred Reporting Items for Systematic Reviews and Meta-Analyses (PRISMA) guideline, with the aim of evaluating the impact of CI on QoL in patients with ARHL (Cumpston et al., 2019). We systematically searched CNKI, PubMed, the Cochrane Library, Embase, and Web of Science from database inception to 7 December 2025. The full search strategies for each database are reported in Supplementary Table S1. All retrieved records were imported into Zotero reference management software, and duplicates were removed. Two reviewers then independently screened titles and abstracts to exclude records that clearly did not meet the eligibility criteria. Full texts of potentially relevant articles were obtained and independently assessed by the same two reviewers (Wxk and Lhx) against pre-specified inclusion and exclusion criteria. Any disagreements were resolved by discussion, with a third reviewer (Lrl) acting as arbiter when consensus could not be reached. The review protocol was prospectively registered in PROSPERO (CRD42024620755).

### 2.2 Inclusion and exclusion criteria

Inclusion criteria: (1) Participants were aged ≥60 years. Hearing loss had to meet a definition of ARHL or be clinically highly consistent with the typical presbycusis phenotype: post-lingual onset; bilateral, relatively symmetrical, progressive sensorineural hearing loss; an audiometric profile dominated by high-frequency sensorineural loss; and no clear alternative etiology such as substantial noise exposure, ototoxic medication, hereditary causes, or middle ear disease. For mixed-etiology cohorts, we included studies only when data for an older subgroup fulfilling this ARHL definition could be extracted separately. (2) Intervention: participants received cochlear implantation (CI), with no restrictions on laterality (unilateral or bilateral), timing of implantation, device model, or speech-processing strategy. (3) Outcomes: studies reported at least one outcome derived from a validated QoL instrument. These included, but were not limited to, hearing/CI-specific questionnaires such as NCIQ, GBI, and International Outcome Inventory for Cochlear Implants (IOI-CI), and generic or health-related QoL instruments such as SF-36, EuroQol 5-Dimension (EQ-5D), HUI2/HUI3, and WHOQOL/WHOQOL-OLD. (4) Study design: prospective or retrospective cohort studies and clinical follow-up studies. (5) Publication: full-text, peer-reviewed original articles published in Chinese or English.

Studies were excluded if they met any of the following criteria: (1) Non-ARHL etiologies, including otosclerosis; chronic otitis media/cholesteatoma; Ménière’s disease; trauma (e.g., blast injury, temporal bone fracture); noise-induced hearing loss; meningitis or other infection-related hearing loss; hereditary or congenital hearing loss; drug-induced ototoxic hearing loss; unilateral sensorineural hearing loss; or mixed-etiology cohorts in which an ARHL-defined older subgroup could not be isolated. (2) Ineligible designs, including case reports, narrative reviews, systematic reviews or meta-analyses, expert opinion pieces, conference abstracts, and theses/dissertations. (3) Inadequate outcome reporting: no QoL-related outcomes; use of unvalidated QoL instruments; or data available only in graphical form without sufficient information to extract or calculate effect sizes. (4) Limited accessibility: full text not obtainable, or publication in languages other than Chinese or English.

### 2.3 Data extraction and risk of bias assessment

Two reviewers (Wxk and Lhx) independently extracted data and assessed risk of bias. Any disagreements were resolved by discussion, with a third reviewer (Lrl) consulted to reach consensus when needed. Risk of bias in non-randomized studies was evaluated using the Cochrane ROBINS-I tool (Sterne et al., 2016).

## 3 Results

### 3.1 Literature search

**Figure 1:**
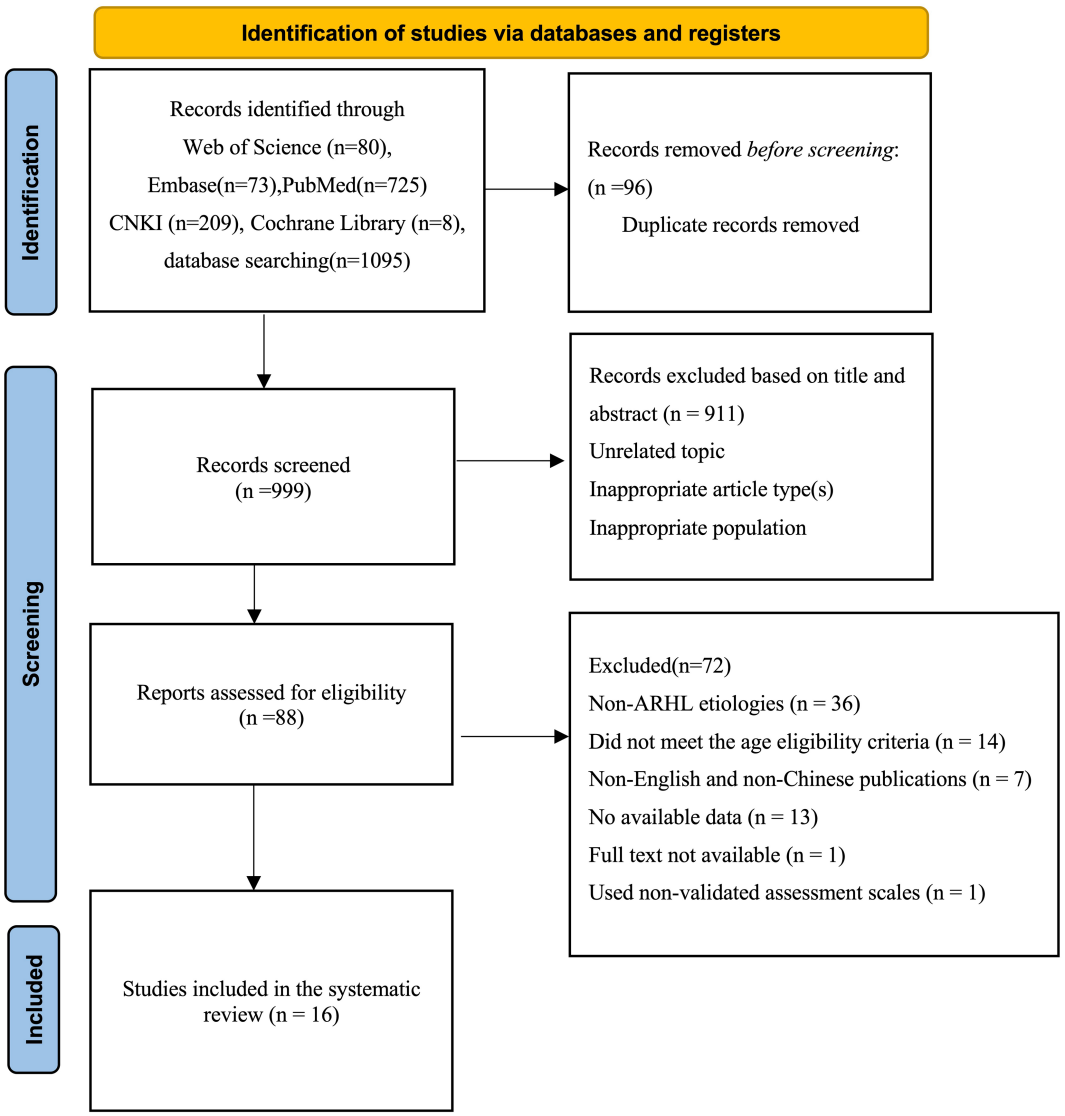
PRISMA 2020 flow diagram of study selection.

The database search yielded 1,095 records, of which 16 studies met the inclusion criteria (Olze et al., 2012, 2016; Ramos et al., 2013; Knopke et al., 2016; Manrique-Huarte et al., 2016; Tang et al., 2017; Mosnier et al., 2018; Issing et al., 2020, 2022, 2024; Sorrentino et al., 2020; Amin et al., 2021; Liu et al., 2021; Knopke et al., 2019; Völter et al., 2020; Li et al., 2024). The PRISMA flow diagram of study selection is presented in Figure 1.

### 3.2 Characteristics of included studies

The 16 included studies were conducted predominantly in Germany, China, and Spain and together enrolled 842 participants. CI was most commonly performed unilaterally, and follow-up ranged from 1 month to 10 years after implantation. Key study characteristics are presented in Table 1, and preoperative audiological criteria together with key inclusion and exclusion criteria are provided in Supplementary Table S2.

**Table 1:** Basic characteristics of the included studies.

Study (year)	Country	Study design	Age, years (mean ± SD or range)	Age groups, years	Sample size	CI configuration	Follow-up schedule	Outcome measures
Li et al. (2024)	China	Retrospective cohort study	67.2 ± 5.6 (60–77)	≥60	31	Unilateral	12 months	NCIQ
Knopke et al. (2019)	Germany	Prospective cohort study	76.2 ± 5.3 (70–88.9)	70–79; ≥80	86	Unilateral	6; 12 months	NCIQ
Völter et al. (2020)	Germany	Prospective cohort study	72.3 ± 5.3 (65–84)	≥65	41	Unilateral	12 months	NCIQ
Olze et al. (2016)	Germany	Prospective cohort study	75.4 (70–88)	≥70	79	Unilateral	6 months	NCIQ
Ramos et al. (2013)	Spain	Retrospective cohort study	69.0 ± 6.6	60–69; ≥70	26	Unilateral (11 cases with a hearing aid on the non-implanted ear)	≥6 months	GBI
Sorrentino et al. (2020)	Italy	Prospective cohort study	Median 71 (IQR 67–75)	≥65	25	23 unilateral; 2 bilateral	≥12 months	GBI
Issing et al. (2022)	Germany	Prospective cohort study	75.3 ± 7.3 (65–101)	≥65	84	Unilateral	1–3; 4–6; 7–10 years	WHOQOL-OLD
Tang et al. (2017)	United States	Mixed design	75.4 ± 6.5 (65–87)	65–74; ≥75	33	Unilateral	12 months	GBI
Amin et al. (2021)	UK	Retrospective cohort study	76.4 ± 5.3 (70.0–90.1)	70–79; ≥80	64	Unilateral	≥12 months	GBI
Manrique-Huarte et al. (2016)	Spain	Mixed design	77.0 ± 6.2	≥65	45	Unilateral	≥ 24 months	HUI3
Mosnier et al. (2018)	France	Multicenter observational cohort study	72.0 ± 5.1 (65–85)	65–74; ≥75	70	Unilateral	12;75 months	NCIQ
Issing et al. (2020)	Germany	Prospective cohort study	73.5 ± 4.9 (65–86)	≥65	34	Unilateral	1; 6 months	WHOQOL-OLD
Liu et al. (2021)	China	Retrospective cohort study	76.8 ± 8.5	61–70; 71–80; >80	120	Unilateral	3; 6; 9; 12 months	WHOQOL-OLD
Issing et al. (2024)	Germany	Prospective cohort study	79.1 ± 4.8 (71–92)	≥65	31	Mainly unilateral CI (some patients converted to bilateral CI during follow-up)	6; 72 months	WHOQOL-OLD
Olze et al. (2012)	Germany	Retrospective cohort study	74.4 ± 4.6 (70–84)	≥70	20	Unilateral	≥6 months	NCIQ; SF-36
Knopke et al. (2016)	Germany	Prospective cohort study	82.9 ± 2.7 (80–101)	≥80	17	Unilateral	6 months	NCIQ

NCIQ, Nijmegen Cochlear Implant Questionnaire; GBI, Glasgow Benefit Inventory; WHOQOL-OLD, World Health Organization Quality of Life – Old; HUI3, Health Utilities Index Mark 3; SF-36, 36-Item Short Form Health Survey.

In Issing et al. (2022), outcomes were analyzed according to three follow-up intervals: 1–3, 4–6, and 7–10 years.

Mixed design (retrospective baseline with prospective follow-up).

### 3.3 Risk of bias

ROBINS-I ratings for individual studies are summarized in Table 2. Overall, risk of bias ranged from moderate to serious, with 37.5% (6/16) of studies classified as having a serious risk of bias. Recurrent concerns included incomplete control of key confounders (e.g., age, multimorbidity, preoperative cognitive status), possible selection bias in participant inclusion, outcome measurement relying mainly on self-administered questionnaires without blinding, and loss to follow-up with limited handling of missing data.

**Table 2:** Risk of bias assessment for non-randomized studies using the ROBINS-I tool.

Study (year)	Bias due to confounding	Bias in selection of participants into the study	Bias in classification of interventions	Bias due to deviations from intended interventions	Bias due to missing data	Bias in measurement of outcomes	Bias in selection of the reported result	Overall risk of bias
Li et al. (2024)	Serious	Low	Low	Moderate	Low	Moderate	Moderate	Serious
Knopke et al. (2019)	Moderate	Low	Low	Low	Moderate	Moderate	Low	Moderate
Völter et al. (2020)	Moderate	Low	Low	Low	Moderate	Moderate	Moderate	Moderate
Olze et al. (2016)	Serious	Moderate	Low	Low	Low	Moderate	Low	Serious
Liu et al. (2021)	Moderate	Low	Low	Moderate	Serious	Moderate	Serious	Serious
Issing et al. (2024)	Moderate	Moderate	Low	Moderate	Moderate	Moderate	Moderate	Moderate
Olze et al. (2012)	Moderate	Serious	Low	Moderate	Serious	Moderate	Moderate	Serious
Knopke et al. (2016)	Moderate	Low	Low	Low	Low	Moderate	Low	Moderate
Ramos et al. (2013)	Moderate	Moderate	Low	Moderate	Moderate	Moderate	Moderate	Moderate
Sorrentino et al. (2020)	Moderate	Moderate	Low	Moderate	Low	Moderate	Moderate	Moderate
Issing et al. (2022)	Moderate	Low	Low	Low	Moderate	Moderate	Moderate	Moderate
Tang et al. (2017)	Moderate	Serious	Low	Moderate	Serious	Moderate	Moderate	Serious
Amin et al. (2021)	Serious	Serious	Low	Moderate	Moderate	Moderate	Moderate	Serious
Manrique-Huarte et al. (2016)	Moderate	Moderate	Low	Moderate	Moderate	Moderate	Moderate	Moderate
Mosnier et al., 2018	Moderate	Moderate	Low	Low	Moderate	Moderate	Moderate	Moderate
Issing et al., 2020	Moderate	Moderate	Low	Low	Low	Moderate	Moderate	Moderate

### 3.4 Narrative synthesis

#### 3.4.1 Hearing/CI-specific instruments

Seven studies used the NCIQ to evaluate QoL and consistently reported that overall QoL improved after CI in patients with ARHL compared with preoperative levels. Across studies, total NCIQ scores increased clearly after implantation at both 6 and 12 months. In the cohort reported by Knopke et al., gains at 12 months exceeded those observed at 6 months (Knopke et al., 2019). Subscale analyses indicated that basic and advanced sound perception domains showed the largest and most stable improvements over time. In contrast, findings from the same research group suggested only a trend toward improvement in the speech production domain at 6- and 12-month follow-up (Knopke et al., 2016, 2019). Mosnier et al. found no significant differences in NCIQ subscale scores between 1 and 7 years after implantation, suggesting that early disease-specific benefits can be sustained over long-term follow-up (Mosnier et al., 2018). With regard to age, most studies did not detect significant between-group differences in the magnitude of NCIQ improvement. One exception was the study by Olze et al., which reported larger QoL gains in patients aged >70 years (Olze et al., 2012).

Four studies applied the GBI to assess QoL after CI in ARHL, all showing higher total GBI scores postoperatively, consistent with high overall satisfaction and perceived benefit from CI. At the subscale level, most studies reported marked gains in the general benefit domain, whereas changes in social support and physical health were less consistent. Ramos et al. specifically noted that the social support domain showed the smallest improvement (Ramos et al., 2013). Most studies did not identify age-related differences in GBI change scores; however, Ramos et al. reported a strong positive correlation between total GBI score and age (*R* = 0.95).

#### 3.4.2 Generic QoL instruments

Four studies employed the WHOQOL-OLD to assess QoL in ARHL, all reporting improvements in overall QoL after CI. In a series of studies from the same group (Issing et al., 2020, 2022, 2024), total WHOQOL-OLD scores at 1 month did not differ significantly from baseline but were significantly higher at 6 months postoperatively. Subscale analyses revealed that sensory abilities temporarily declined at 1 month relative to baseline but showed significant improvement by 6 months. Social participation also improved significantly at 6 months, whereas other domains did not change significantly. Long-term follow-up data indicated that, 6 years after implantation, total WHOQOL-OLD scores and all subscale scores remained significantly higher than preoperative values, with no significant differences in improvement across follow-up strata of 1–3, 4–6, and 7–10 years. These findings suggest that early overall QoL gains are largely maintained over the medium to long term. Another study reported a continuous upward trajectory in total QoL scores with longer follow-up, with the largest gain occurring around 9 months after CI, and greater benefits observed in younger members of the older cohort (Liu et al., 2021).

For other generic instruments, a study using the HUI3 found that overall health utility in CI users was significantly higher than in untreated individuals with severe sensorineural deafness and comparable to that of hearing aid users (Manrique-Huarte et al., 2016). A study using the SF-36 showed that, after CI, social functioning and mental health improved in older adults but to a lesser degree than in younger adults. In contrast, scores for physical functioning and bodily pain in older adults tended to decline over time during follow-up.

## 4 Discussion

Drawing on a body of evidence composed predominantly of observational cohort studies, this review indicates that CI is generally associated with improved QoL in patients with ARHL. The most consistent benefit signals were observed in hearing/CI-specific instruments such as the NCIQ and GBI, whereas generic instruments more often showed delayed improvements, predominantly in social function–related domains.

The QoL constructs examined here can broadly be grouped into two categories. The first is social functioning, referred to as “social participation/social interaction” and encompassing NCIQ social interaction, GBI social support, WHOQOL-OLD social participation, and SF-36 social functioning. The second is physical health, including GBI physical health and SF-36 physical functioning and bodily pain. Other domains are reported using their original labels as defined in each instrument. The coherent improvement patterns observed for NCIQ and GBI likely reflect the close alignment between what these instruments measure and the primary mechanisms of CI benefit. Hearing/CI-specific instruments focus on functions directly targeted by CI—such as sound perception and social functioning—and are therefore particularly sensitive to changes in auditory and communication outcomes. By contrast, generic instruments such as the SF-36 and WHOQOL-OLD include several physical health domains that are only indirectly related to hearing interventions and are strongly shaped by comorbidity and normal aging in older adults, contributing to more variable trajectories in these scores (Andries et al., 2021). Consistent with our findings, a prospective multicenter study reported that, within the same CI cohort, hearing/CI-specific instruments were more sensitive than generic measures during follow-up (Lassaletta et al., 2022). The GBI, which explicitly captures pre–post change, is well suited to detecting general benefit and social function–related improvement. While reliable and useful in CI populations, it may be best positioned as a complementary indicator of perceived benefit (Lui et al., 2021). More recently, CI-specific instruments such as the Cochlear Implant Quality of Life (CIQOL) suite have been developed to more precisely map key CI benefit domains—communication, emotion, entertainment, environment, listening effort, and social situations—onto patients’ lived experience (McRackan et al., 2019). In comparative studies, the CIQOL-35 and CIQOL-10 have shown stronger construct validity and more robust reliability than several legacy tools, highlighting limitations in NCIQ structural validity and weaker reliability of the HUI3 (McRackan et al., 2021a). Ongoing work is extending CIQOL through cross-cultural adaptation and localization (Laplante-Lévesque et al., 2021; Pützer et al., 2024). Overall, hearing/CI-specific instruments appear to capture early, direct gains in sound perception, whereas generic instruments are better suited to reflecting the downstream social and psychological benefits that accrue with device acclimatization, listening training, and changes in everyday communication behavior. Future CI studies in older adults should therefore prioritize hearing/CI-specific instruments and CIQOL as primary outcome measures, using generic QoL instruments mainly as complementary endpoints, particularly for long-term follow-up.

At the domain level, we found that NCIQ improvements were most pronounced and stable over time in the basic and advanced sound perception domains, consistent with the direct impact of CI on sound detection, sound localization, and listening in complex auditory environments. Prior work has shown that bilateral CI, compared with unilateral implantation, can further enhance sound localization and spatial hearing (Gaylor et al., 2013). Importantly, however, gains in speech recognition do not automatically translate into parallel improvements in QoL (McRackan et al., 2018). In studies using the WHOQOL-OLD, a characteristic pattern emerged in which no significant changes were seen shortly after surgery, whereas significant improvements were observed at 6 months, particularly in sensory abilities and social functioning (Issing et al., 2020, 2022, 2024). This pattern suggests that overall QoL gains detected by generic instruments typically consolidate only over time. They become more apparent after patients have undergone device adaptation, programming optimization, auditory training, and reorganization of daily communication patterns. By contrast, physical health domains in generic instruments primarily reflect cardiopulmonary fitness, musculoskeletal status, neurodegeneration, and chronic disease burden; from a mechanistic perspective, they are not expected to improve to the same extent or pace as hearing-related outcomes. Furthermore, prior reports indicate that postoperative complications after CI are most often mild, self-limited local or vestibular problems—such as incision or skin discomfort, postoperative vertigo, and imbalance—whereas serious complications requiring revision surgery or leading to marked deterioration in overall health are relatively uncommon (Kanai et al., 2021; Orlando and Cruz, 2024). In very old recipients with multiple chronic conditions, such transient vestibular and somatic discomfort is not uncommon in the early postoperative period and may restrict daily activities and participation outside the home during recovery, thereby temporarily lowering scores in physical health–related domains of generic instruments at short-term follow-up (Vaz et al., 2022; Kutlu et al., 2024). Previous studies have also reported that overall QoL in older CI users can approximate that of healthy age-matched controls, while domains such as social functioning are more sensitive to CI-related change (Tokat et al., 2021). In terms of trajectory, QoL tends to improve over 6–12 months postoperatively (Lassaletta et al., 2022), with delayed effects such as increased everyday independence and reduced loneliness emerging beyond 18 months (Cuda et al., 2024). This pattern supports the view that CI influences QoL through a stepwise process, in which early auditory gains gradually extend to social and emotional domains. Beyond QoL, improved hearing and communication after CI may also indirectly affect broader health-related outcomes, including healthcare utilization, work ability, and social functioning (Bekele Okuba et al., 2023). Rehabilitation interventions such as auditory training have been shown to further enhance auditory and functional performance (Dornhoffer et al., 2024), underscoring that CI care should extend beyond device programming to include structured auditory training and strategies to promote social engagement. Consequently, preoperative counseling and follow-up assessments should emphasize realistic expectation management, avoid attributing changes in physical function solely to CI efficacy, and prioritize social functioning and communication opportunities as key follow-up and intervention targets. Enhancing family support, creating social participation opportunities, and providing rehabilitation training may help amplify and stabilize QoL benefits.

In our synthesis, most studies reported similar QoL gains across age groups, and a few even suggested larger improvements among patients aged ≥70 years. This pattern may reflect lower baseline QoL and more restricted social functioning in the very old, creating greater room for improvement, while perceived benefit from restoring basic hearing and communication may be comparable between very old and “younger old” adults. Age therefore seems to shape the conditions under which benefit can be achieved and sustained rather than determining whether benefit is possible. Recent evidence shows that recipients aged ≥80 years can still achieve meaningful improvements in auditory outcomes, although sustained device use may decrease over time. Discontinuation is typically driven by declining independence, challenges in device handling, and limited support networks rather than by an absence of auditory benefit in very old age (Hallin et al., 2023; Bocquillon et al., 2025). Data from the German national CI registry also point to under-treatment or incomplete coverage among adults aged ≥85 years, suggesting a need to improve access for very old candidates when expected benefit is clear (Stöver et al., 2024). Furthermore, significant postoperative improvements in speech recognition have been documented in recipients aged 80–89 and ≥90 years, reinforcing that very old adults can obtain substantial auditory gains (Zhan et al., 2025). Collectively, these data suggest that advanced age per se should not be viewed as an absolute barrier to CI; instead, candidacy decisions should be guided by a holistic assessment of overall health status.

This review has several limitations. First, studies used a mix of hearing/CI-specific and generic instruments with different domain structures and follow-up durations ranging from 1 month to several years, limiting direct comparability of outcomes across studies. Second, all included studies were observational, and ROBINS-I ratings indicated moderate to serious risk of bias. Many studies did not systematically adjust for key confounders, such as educational attainment, comorbidities, and participation in rehabilitation; consequently, the overall certainty of the evidence is low. Finally, although all included studies targeted adults with ARHL, the operational definitions of ARHL and the level of detail with which key exclusion criteria were reported varied across studies. As a result, residual mixed etiologies cannot be entirely ruled out, which limits the interpretation and generalizability of QoL-related conclusions.

## 5 Conclusion

This systematic review suggests that CI is generally associated with improved QoL in patients with ARHL. However, this conclusion is supported primarily by observational studies that are at moderate to serious risk of bias, particularly with respect to confounding and participant selection, so the overall certainty of the evidence is low. Benefit signals were most sensitive and internally consistent in hearing/CI-specific instruments such as the NCIQ and GBI, particularly in core domains related to sound perception and social functioning. Generic instruments (e.g., WHOQOL-OLD, SF-36, HUI3) more often revealed delayed and domain-specific gains, mainly in sensory abilities, social functioning, and mental health, while physical-function domains showed only limited improvement, likely reflecting the influence of aging and multimorbidity. Overall, current evidence does not support chronological age per se as a major constraint on CI-related benefit. Future studies should adopt more rigorous prospective designs, harmonize follow-up schedules and outcome frameworks, and prioritize CI-specific standardized tools (such as CIQOL) to enhance consistency of outcome measurement and provide more robust data on CI benefit across ARHL populations of different ages.

## Data Availability

The original contributions presented in the study are included in the article/Supplementary material, further inquiries can be directed to the corresponding author.
