# Supplementary material for: Effects of cochlear implantation on quality of life in patients with age-related hearing loss: a systematic review
Source: Front Neurosci. 2026 Feb 25;20:1778985. doi: 10.3389/fnins.2026.1778985 (PMC12975979; doi:10.3389/fnins.2026.1778985)
Supplement: Supplementary file 1 [file Table_1.docx]

**Effects of cochlear implantation on quality of life in patients with age-related hearing loss: a systematic review**

| database | search strategy |
| --- | --- |
| pubmed: | ("presbycusis"[All Fields] OR "presbyacusis"[All Fields] OR "age-related hearing loss"[All Fields] OR "elderly hearing loss"[All Fields] OR "age-associated hearing loss"[All Fields] OR "age-related hearing impairment"[All Fields] OR "age-related deafness"[All Fields] OR "ARHL"[All Fields] OR (("hearing loss"[MeSH Terms] OR ("hearing"[All Fields] AND "loss"[All Fields]) OR "hearing loss"[All Fields] OR ("hearing"[All Fields] AND "impairment"[All Fields]) OR "hearing impairment"[All Fields]) AND ("aged"[MeSH Terms] OR "aged"[All Fields] OR "elderly"[All Fields] OR "elderlies"[All Fields] OR "elderly s"[All Fields] OR "elderlys"[All Fields]))) AND ("cochlear implant"[All Fields] OR "cochlear implants"[All Fields] OR "cochlear implantation"[All Fields] OR "CI"[All Fields]) AND ("quality of life"[All Fields] OR "health-related quality of life"[All Fields] OR "HRQOL"[All Fields] OR "QOL"[All Fields] OR "life satisfaction"[All Fields] OR "SF-36"[All Fields] OR "EQ-5D"[All Fields] OR "WHOQOL"[All Fields] OR "NCIQ"[All Fields] OR "GBI"[All Fields] OR "HUI"[All Fields] OR "HUI2"[All Fields] OR "HUI3"[All Fields] OR "HUI-2"[All Fields] OR "HUI-3"[All Fields] OR ("SSQ"[All Fields] OR "SSQ12"[All Fields] OR "SSQ-B"[All Fields]) OR "HHIE-S"[All Fields] OR "HHIE"[All Fields] OR "IOI-CI"[All Fields] OR "HHIA"[All Fields] OR "HHIA-S"[All Fields]) |
| Cochrane Library： | #1 ("presbycusis") OR ("presbyacusis") OR ("age-related hearing loss") OR ("elderly hearing loss") OR ("age-associated hearing loss") (Word variations have been searched) 150  #2 ("age-related hearing impairment") OR ("age-related deafness") OR ("ARHL") OR ("Hearing impairment in elderly") (Word variations have been searched) 26  #3 ("cochlear implant") OR ("cochlear implants") OR ("cochlear implantation") OR ("CI") (Word variations have been searched) 181752  #4 ("quality of life") OR ("health-related quality of life") OR ("HRQOL") OR ("QOL") OR ("life satisfaction") (Word variations have been searched) 195941  #5 ("SF-36") OR ("EQ-5D") OR ("WHOQOL") OR ("NCIQ") OR ("GBI") (Word variations have been searched) 32261  #6 ("HUI") OR ("HUI2") OR ("HUI3") OR ("HUI-2") OR ("HUI-3") (Word variations have been searched) 2764  #7 ("SF-12") OR ("SSQ") OR ("SSQ12") OR ("SSQ-B") OR ("HHIE-S") 4153  #8 ("HHIE") OR ("IOI-CI") OR ("HHIA") OR ("HHIA-S") 59  #9 #1 OR #2 152  #10 #4 OR #5 OR #6 OR #7 OR #8 206139  #11 #9and#3and#10 8 |
| web of science： | 1: ((((((((ALL=("presbycusis")) OR ALL=("presbyacusis")) OR ALL=("age-related hearing loss")) OR ALL=("elderly hearing loss")) OR ALL=("age-associated hearing loss")) OR ALL=("age-related hearing impairment")) OR ALL=("age-related deafness")) OR ALL=("ARHL")) OR ALL=("Hearing impairment in elderly") Results: 3226  3: (((ALL=("cochlear implant")) OR ALL=("cochlear implants")) OR ALL=("cochlear implantation")) OR ALL=("CI")  Results: 1286421  5: ((((((((((((((((((((((ALL=("quality of life")) OR ALL=("health-related quality of life")) OR ALL=("HRQOL")) OR ALL=("QOL")) OR ALL=("life satisfaction")) OR ALL=("SF-36")) OR ALL=("EQ-5D")) OR ALL=("WHOQOL")) OR ALL=("NCIQ")) OR ALL=("GBI")) OR ALL=("HUI")) OR ALL=("HUI2")) OR ALL=("HUI3")) OR ALL=("HUI-2")) OR ALL=("HUI-3")) OR ALL=("SSQ")) OR ALL=("SSQ12")) OR ALL=("SSQ-B")) OR ALL=("HHIE-S")) OR ALL=("HHIE")) OR ALL=("IOI-CI")) OR ALL=("HHIA")) OR ALL=("HHIA-S") Results: 1292273  6: #1 AND #3 AND #5 Results: 80 |
| Embase： | #4. #1 AND #2 AND #3 73  #3. 'cochlear implant'/exp OR 'cochlear implant' OR 1,819,480  'cochlear implants' OR 'cochlear implantation' OR  'ci'  #2. 'quality of life'/exp OR 'quality of life' OR 1,460,802  'health-related quality of life' OR 'hrqol' OR  'qol' OR 'life satisfaction' OR 'sf-36' OR  'eq-5d' OR 'whoqol' OR 'nciq' OR 'gbi' OR 'hui'  OR 'hui2' OR 'hui3' OR 'hui-2' OR 'hui-3' OR  'ssq' OR 'ssq12' OR 'ssq-b' OR 'hhie-s' OR 'hhie'  OR 'ioi-ci' OR 'hhia' OR 'hhia-s'  #1. 'presbycusis'/exp OR 'presbycusis' OR 4,751  'presbyacusis' OR 'age-related hearing loss' OR  'elderly hearing loss' OR 'age-associated hearing  loss' OR 'age-related hearing impairment' OR  'age-related deafness' OR 'arhl' OR 'hearing  impairment in elderly' |
