# Supplementary material for: Effects of cochlear implantation on quality of life in patients with age-related hearing loss: a systematic review
Source: Front Neurosci. 2026 Feb 25;20:1778985. doi: 10.3389/fnins.2026.1778985 (PMC12975979; doi:10.3389/fnins.2026.1778985)
Supplement: Supplementary file 2 [file Table_2.docx]

**Supplementary Table S2. Preoperative audiological criteria and key inclusion/exclusion criteria across included studies**

| **Study (year)** | **HL phenotype** | **Pre-op audiological criteria (P/C/B) *** | **Key inclusion conditions** | **Key exclusion conditions** |
| --- | --- | --- | --- | --- |
| **Li et al. (2024)** | Profound, SNHL | **P:** PTA > 80 dB HL;  **C:** open-set short-sentence recognition < 40% (HA-aided; quiet; 70 dB SPL);  **B:** Mandarin monosyllable recognition = 9.55% at 70 dB SPL (quiet sound field) | MMSE > 26; hearing-aid use ≥ 6 months preoperatively; met adult CI guideline requirements (audiological + imaging); poor benefit from hearing aids | Pre-anesthesia and multidisciplinary assessment to exclude systemic surgical risks (e.g., cardio-/cerebrovascular risk) |
| **Knopke et al. (2019)** | Severe-to-profound, SNHL | **P:** NA;  **C:** Freiburg monosyllabic speech recognition ≤ 40% at 65 dB SPL (quiet; optimized HA);  **B:** Freiburg at 65 dB SPL = 6.65% | Age ≥ 70; met clinical CI candidacy; eligible for general anesthesia; unremarkable cochlear anatomy; motivation for post-op audiological rehabilitation | Retrocochlear pathology (e.g., vestibular schwannoma) |
| **Völter et al. (2020)** | Post-lingual SNHL, Severe-to-profound | **P:** PTA (0.5/1/2/4 kHz, better ear) ≥ 61 dB HL;  **C:** NA;  **B:** Freiburg monosyllables at 65 dB SPL (free field; quiet) = 7.5% | Native-level German proficiency; no global cognitive impairment (MWT-B); no history of severe depression; no CNS disease; no anticholinergic medication use | NA |
| **Olze et al. (2016)** | SNHL | **P:** NA;  **C:** Freiburg monosyllabic recognition ≤ 40% at 65 dB SPL (quiet; optimized hearing aids);  **B:** Freiburg at 65 dB SPL (quiet) = 6.73% | Age ≥ 70 at implantation | NA |
| **Ramos et al. (2013)** | Severe HL | **P:** Audiometric threshold 91.12 ± 33.2 dB HL (aided in HA users; as reported);  **C:** NA**;**  **B:** NA**;** | Single-center CI cohort; complete follow-up data available | NA |
| **Sorrentino et al. (2020)** | Severe progressive post-lingual HL | **P**: NA;  **C**: NA;  **B:** sentence recognition, median (IQR) 0 (0–20), pre-CI in quiet | CI use ≥ 1 year; regular CI fitting sessions; speech therapy for 1 year | NA |
| **Issing et al. (2022)** | profound HL | **P**: NA;  **C**: NA;  **B**: Freiburg monosyllabic speech test (FMS) at 65 dB SPL: I:15.3 ± 19.3 / II: 16.9 ± 24.7 / III: 9.6 ± 12.3% (subgroups); free field; best-aided; contralateral masking | Unilateral CI use ≥ 1 year and ≤ 10 years; native-level German proficiency | Known dementia; psychiatric disorders (e.g., major depression, psychotic disorders) |
| **Tang et al. (2017)** | Severe-to-profound post-lingual HL | **P**: NA;  **C**: NA;  **B**: AzBio sentences at 60 dB SPL = 60.2% | Age ≥ 65 at implantation | Onset of deafness ≤ 3 years of age; bilateral CI; need for a language interpreter |
| **Amin et al. (2021)** | Not explicitly specified (CI candidacy based on contemporaneous NICE audiological profile) | **P**: NA;  **C**: NA;  **B**: BKB sentences at 70 dB SPL (quiet) score not reported (NA). | Age ≥ 70; consecutive unilateral CI recipients at St. Thomas’ Hearing Implant Centre (London, UK; 2008–2017); follow-up ≥ 12 months | NA |
| **Manrique-Huarte et al. (2016)** | Severe-to-profound, SNHL | **P**: mean PTA = 99.6 dB HL;  **C**: NA;  **B**: Spanish disyllabic words at 65 dB SPL (quiet) = 1.0% | Complete pre-implant and ≥ 2-year post-treatment data | Pfeiffer test errors < 3 (i.e., no severe cognitive impairment) |
| **Mosnier et al. (2018)** | Severe-to-profound post-lingual HL | **P**: NA;  **C**: Open-set disyllabic words (Fournier), quiet, best-aided (verified optimal HA fitting), 60 dB SPL, ≤50%.  **B**: Speech perception in quiet (HA only) =1.3%. | Age ≥ 65 at implantation; follow-up ≥ 5 years for each participant; native French speakers | NA |
| **Issing et al. (2020)** | profound HL | **P**: NA;  **C**: NA;  **B:** Freiburg monosyllables (FMS) at 65 dB SPL = 14.7 ± 19.9%; free field; best-aided; contralateral masking | First-time unilateral CI surgery planned | Dementia screening (DemTect) used to exclude dementia; GDS used to exclude depression |
| **Liu et al. (2021)** | Severe SNHL | **P**: Aided PTA (0.5/1/2/4 kHz) = 48.8 ± 9.2 dB HL (with HA);  **C**: NA;  **B**: NA; | Postoperative adaptation judged as satisfactory (as per original study selection). | Organic etiologies (e.g., Ménière’s disease, vestibular schwannoma, nasopharyngeal carcinoma); psychiatric disorders; stroke/Parkinson’s disease/dementia; incomplete data or poor follow-up adherence |
| **Issing et al. (2024)** | profound HL | **P**: NA;  **C**: NA;  B: Freiburg monosyllables (FMS) at 65 dB SPL = 14.7 ± 19.9%; free field; best-aided; contralateral masking | First-time unilateral CI recipients; annual postoperative follow-up for ~6 years. | Follow-up attrition: explantation due to implant infection (n=1); non-CI-related health problems preventing participation (n=2). |
| **Olze et al. (2012)** | SNHL | **P**: NA;  **C**: Freiburg monosyllabic recognition ≤ 40% at 65 dB SPL (quiet; optimized hearing aids);  **B**: Freiburg at 65 dB SPL = 2.5% | Appropriately fitted/optimized hearing aids | NA |
| **Knopke et al. (2016)** | Progressive post-lingual HL | **P**: NA;  **C**: Freiburg monosyllabic recognition ≤ 40% at 65 dB SPL (quiet; optimized hearing aids);  **B**: Freiburg at 65 dB SPL = 3.2 ± 8.5% | Appropriately fitted/optimized hearing aids | NA |

Note:

(1) * P/C/B definition for harmonization: P = pure-tone/audiometric threshold criterion (including PTA where reported); C = candidacy “gatekeeping” speech criterion used for CI eligibility; B = baseline preoperative speech perception score reported for the implanted cohort (or relevant subgroup), as available.

(2) Abbreviations: CI, cochlear implant; HA, hearing aid; PTA, pure-tone average; SPL, sound pressure level; SR, sentence recognition; FMS, Freiburg Monosyllabic Speech Test; BKB, Bamford–Kowal–Bench; MWT-B, Multiple Word Sentence Test; MMSE, Mini-Mental State Examination; GDS, Geriatric Depression Scale.

(3) In Issing et al. (2022), outcomes were analyzed across three follow-up intervals: Group I (1–3 years), II (4–6 years), and III (7–10 years).

(4) In some studies, preoperative ‘thresholds’ were reported under aided conditions; we reproduced the original reporting without conversion.

(5)NA=indicates not reported. Quiet = speech testing performed in quiet conditions. Free-field = sound-field presentation via loudspeaker.
